# AFM-Detected Apoptotic Changes in Morphology and Biophysical Property Caused by Paclitaxel in Ishikawa and HeLa Cells

**DOI:** 10.1371/journal.pone.0030066

**Published:** 2012-01-17

**Authors:** Kyung Sook Kim, Chang Hoon Cho, Eun Kuk Park, Min-Hyung Jung, Kyung-Sik Yoon, Hun-Kuk Park

**Affiliations:** 1 Department of Biomedical Engineering, College of Medicine, Kyung Hee University, Seoul, Korea; 2 Healthcare Industry Research Institute, Kyung Hee University, Seoul, Korea; 3 Department of Biochemistry and Molecular Biology, College of Medicine, Kyung Hee University, Seoul, Korea; 4 Department of Medical Zoology, College of Medicine, Kyung Hee University, Seoul, Korea; 5 Division of Gynecologic Oncology, College of Medicine, Kyung Hee Medical Center, Kyung Hee University, Seoul, Korea; 6 Program of Medical Engineering, Kyung Hee University, Seoul, Korea; Swiss Federal Institute of Technology Zurich, Switzerland

## Abstract

The apoptosis of cancer cells is associated with changes in the important cell properties including morphology, surface roughness and stiffness. Therefore, the changes in morphology and biophysical properties can be a good way of evaluating the anticancer activity of a drug. This study examined the effect of paclitaxel on the properties of Ishikawa and HeLa cells using atomic force microscopy (AFM), and the relationship between the changes in morphology and the biophysical properties and apoptosis was discussed. The viability and proliferation of the cells were analyzed using the methylthiazol tetrazolium (MTT) method and a TUNEL assay to confirm cellular apoptosis due to a paclitaxel treatment. AFM observations clearly showed the apoptotic morphological and biophysical changes in Ishikawa and HeLa cells. After the paclitaxel treatment, the cell membrane was torn and holed, the surface roughness was increased, and the stiffness was decreased. These changes were observed more apparently after a 24 h treatment and in Ishikawa cells compared to HeLa cells. The MTT and TUNEL assays results revealed the Ishikawa cells to be more sensitive to paclitaxel than HeLa cells and definite apoptosis occurred after a 24 h treatment. These results showed good agreement with the AFM results. Therefore, research on the morphological and biophysical changes by AFM in cancer cells will help to evaluate the anticancer activities of the drugs.

## Introduction

Paclitaxel is an antineoplastic agent that is commonly used in the treatment of human carcinomas [1]–[6] and has shown promising potential in the treatment of epithelial cancers, such as breast, ovarian, lung and colon [1], [2]. As a microtubule-stabilizing agent, the mechanism of action of paclitaxel is considered unique. The drug binds to the β-subunit of tubulin in tumor cells and promotes the formation of stable microtubules. As a result, the dynamic instability of cells decreases and the microtubule rigidity increases, thereby inhibiting cell replication through a disruption of normal mitotic spindle formation [1], [2]. Additional activities of paclitaxel have been reported in a range of tumor cells. Paclitaxel induces apoptosis, which is dependent upon FAS-associated death domain protein through the activation of caspase-10 but is independent of the death receptors [3]. In addition, paclitaxel regulates the expression of the apoptosis-related proteins, such as bcl-2, bad, bcl-xL, and tumor necrosis factor 1 (TNF-α) [4]–[6]. However, the precise mechanism underlying paclitaxel-induced apoptosis in different cell lines and under different stimuli is unclear.

Recently, it was suggested that a study of the morphological and biophysical changes in cancer cells treated with anticancer drugs would help in evaluating the anticancer activity of a drug [7], [8]. This was suggested in part because antimicrotubule drugs affect the shape and physical properties of the cell, such as roughness and stiffness, which are related to the cell functions of adherence, motility, transformation and invasion. These changes in the morphology and physical properties of individual cells can be detected by atomic force microscopy (AFM). Since AFM is a very high-resolution type of scanning probe microscopy, it has been shown to be a powerful tool for imaging materials at the nanometer level and for observing the ultrastructure of a cell [9], [10]. In particular, this method is appropriate for measuring the changes in the biophysical properties of the cell [11], [12]. This research is in the forefront of the field, and few studies have reported the morphological and physical properties of cancer cells after an anticancer drug treatment.

This study evaluated the potential of AFM as a new method for evaluating the anticancer activity of a drug. The effects of paclitaxel on the morphology and biophysical properties of Ishikawa and HeLa cells were examined by AFM. The cell viability and apoptosis were observed using MTT and TUNEL assays, respectively. A correlation was observed between the changes in the morphology and the biophysical property and apoptosis in the cancer cells. These results were discussed in relation to the possible underlying mechanism of action of paclitaxel.

## Results

### 1. Effects of paclitaxel on cell viability as analyzed using the MTT assay

**Figure 1:**
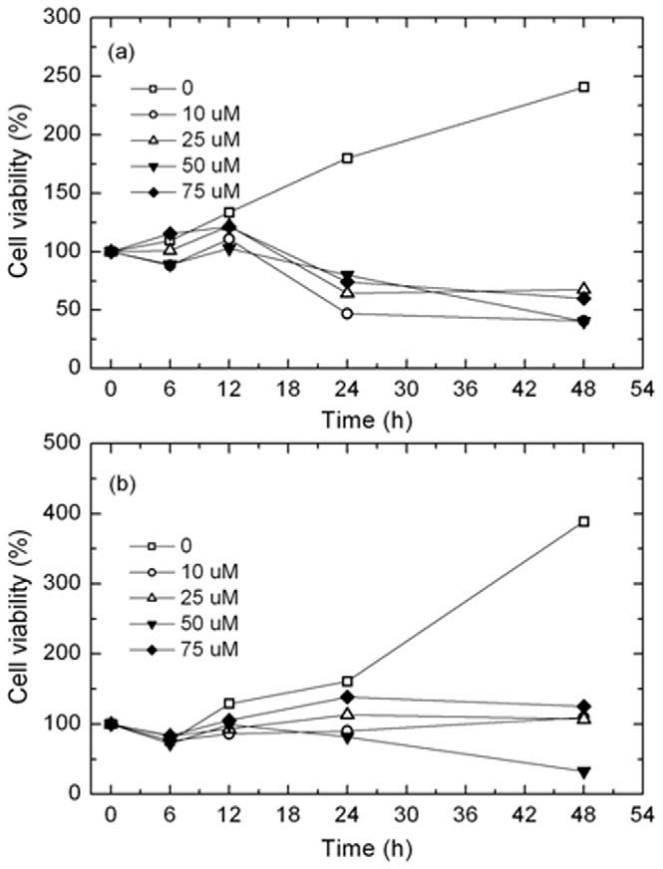
Cell viabilities of Ishikawa (a) and HeLa (b) cells. The cells were seeded (2×10^5^) and treated for various lengths of time (0∼48 h) with various concentrations (10∼75 µM) of paclitaxel and were analyzed using the MTT assay.

The effects of paclitaxel on both Ishikawa and HeLa cells were estimated using a MTT assay, which measures the metabolic activity of the mitochondria. Figures 1(a) and (b) show the rates of Ishikawa and HeLa cell proliferation as a function of the treatment time at different paclitaxel concentrations (DMSO, 10, 25, 50, and 75 µM, respectively). In both cell types, the proliferation rates were increased in DMSO but decreased in paclitaxel. In Ishikawa cells, the proliferation rate decreased significantly in a time- and concentration-dependent manner. The proliferation rates of the Ishikawa cells treated with 10, 25, 50, and 75 µM paclitaxel were -53, -36, -20, and -36% of the baseline, respectively. The assay was performed twice in the same manner. After the MTT assay, 50 µM paclitaxel was used for the TUNEL assay.

### 2. TUNEL assay for apoptotic cells

**Figure 2:**
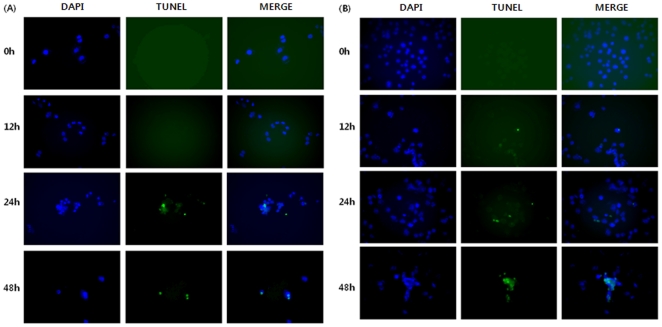
Detection of DNA fragmentation in paclitaxel-treated Ishikawa (A) and HeLa (B) cells using the TUNEL assay.

To evaluate cell death, a TUNEL assay was performed in both Ishikawa and HeLa cells treated with 50 µM paclitaxel for various times (0, 12, 24, and 48 h). All cells were fixed in methanol. Figures 2(A) and (B) show the results of the TUNEL assay of Ishikawa and HeLa cells, respectively. *In situ* apoptosis detections of TdT-Fluor in the control (0 h) and treatment groups (12, 24, and 48 h) were compared to confirm apoptosis and quantify cell death. The TUNEL assay showed more cell death in the treatment groups compared to that in the control group. Large numbers of reactive cells were present in the Ishikawa cells after a 24 h treatment with paclitaxel and in Ishikawa cells after a 12 h paclitaxel treatment. On the other hand, apoptotic cell death was observed in both the Ishikawa and HeLa cells after 24 h of paclitaxel treatment.

### 3. Morphological changes caused by paclitaxel

**Figure 3:**
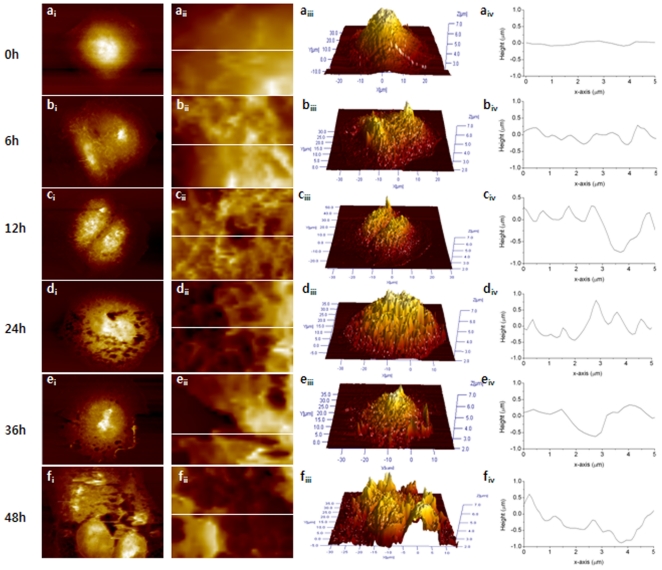
Representative AFM images from both untreated (0 h) and treated Ishikawa cells for 6, 12, 24, 36, and 48 h. The first and second columns show full and magnified images (3×5 µm^2^) of the cell. The third column shows a three dimensional (3D) image of the entire cell and the fourth column is the line profile measured in the magnified image shown in the second column. The white line shows the position taken the line profile. The colors in the images indicate different heights with light and dark colors corresponding to higher and lower topography, respectively.

High-resolution AFM images were obtained for both Ishikawa and HeLa cells fixed in 2.5% GA for 30 min to optimize the imaging conditions. The cells were imaged in PBS buffer solution to avoid any size and shape distortions that would otherwise be caused by drying. Both cell types were treated with 50 µM paclitaxel for various lengths of time. Figure 3 shows representative AFM images of both untreated (0 h) and treated Ishikawa cells for 6, 12, 24, 36 and 48 h.

The untreated Ishikawa cells showed a conventional cellular shape with distinct boundaries and centrally located nuclei. There were no typical morphological changes after 6 h of treatment. On the other hand, when the Ishikawa cells were exposed to paclitaxel for more than 24 h, obvious apoptotic changes, such as aggregation, micronucleated cells and floating cells, were observed. Interestingly, the cell membranes were severely damaged. The membrane of the untreated cell was smooth, as shown in Fig. 3(a_ii_), but was rough and torn after the paclitaxel treatment. The damage became worse as the treatment increased, particularly after 24 h treatment. In addition, the hole appeared on the membrane surface after 12 h of treatment. The hole was small in both number and size and was shallow in depth. The number and depth of the holes increased with increasing treatment time. Detailed information regarding the holes is listed in Table 1.

**Table 1:** Number of holes observed in the cell membrane along with their depths and the cell height as a function of the paclitaxel treatment time in Ishikawa and HeLa cells.

	Hole number (N/cell)		Hole depth (µm)		Cell height (µm)	
Treatment time (h)	Ishikawa	HeLa	Ishikawa	HeLa	Ishikawa	HeLa
0	0	0	0	0	4.11±0.85	4.15±0.70
6	0	0	0	0	3.23±0.71	4.58±1.32
12	8.7±6.70	8.01±2.30	0.63±0.21	0.61±0.21	4.35±1.48	4.43±0.81
24	24.31±6.10	26.01±19.81	1.13±0.59	0.49±0.22	3.81±0.87	3.34±0.50
36	25.01±7.10	14.03±6.80	0.65±0.39	0.83±0.28	3.84±0.14	3.85±0.40
48	20.01±4.10	13.02±5.41	1.72±0.95	0.79±0.44	5.24±1.22	4.46±1.07

The hole and height were measured from 5∼12 cells for each group and the results were normalized by the number of cell. The height of cell was defined as the largest difference between the top and bottom of the cell.

The height of the cell, which was defined as the difference between the top and bottom of the cell, was determined from the 3D images. The height was changed by the paclitaxel treatment, but there was no correlation between the treatment condition and cell height. Table 1 lists the height of Ishikawa cells, which is the averaged data determined from more than 5 cells for each group.

An analysis of the surface roughness provides novel quantitative data for a study of the cancer cell morphology. Table 2 lists the results of two surface roughness parameters, Ra and Rq, determined in an area of 6×6 µm^2^ and 3×3 µm^2^. Both Ra and Rq showed significant dependence on the paclitaxel treatment conditions. The untreated cell showed the smallest Ra and Rq, as expected by the smooth surface shown in Figs. 3(a_ii_) and (a_iv_). In all treated cells, Ra and Rq were more than double (after 24 h) that of the untreated cells indicating a rougher surface, and the values increased with increasing treatment time. The hole is one of source of the rougher surface, as shown in Figs. 3(c_iv_), (d_iv_), (e_iv_), and (f_iv_). The change in roughness in all treated cells was significant compare to the untreated cell.

**Table 2:** The surface roughness parameters Ra and Rq as a function of the paclitaxel treatment time in Ishikawa and HeLa cells.

		Ishikawa cell		HeLa cell	
Area (µm^2^)	Treatment time (*h*)	Ra (p-value)	Rq (p-value)	Ra (p-value)	Rq (p-value)
6×6	0	71.6±20.4	90.8±25.4	92.9±26.7	118.5±34.5
	6	114.2±47.3 (<0.05)	144.3±56.7 (<0.05)	141.7±55.6 (0.02)	180.5±71.9 (0.04)
	12	136.1±43.2 (<0.05)	173.7±51.9 (<0.05)	122.1±37.9 (0.32)	157.9±50.6 (0.14)
	24	193.9±102.9 (<0.05)	247.7±122.4 (<0.05)	97.9±20.6 (0.23)	118.5±28.7 (0.04)
	36	201.4±67.8 (<0.05)	259.8±83.9 (<0.05)	111.3±36.3 (<0.05)	148.3±47.9 (<0.05)
	48	245.8±87.5 (<0.05)	306.5±100.9 (<0.05)	180.1±61.4 (<0.05)	229.2±75.1 (<0.05)
3×3	0	66.4±26.8	85.5±34.5	79.9±23.9	103.3±31.8
	6	115.1±72.9 (<0.05)	144.9±83.2 (<0.05)	96.5±32.4 (0.02)	123.5±42.2 (0.03)
	12	103.6±43.7 (<0.05)	142.9±57.1 (<0.05)	89.6±37.8 (0.37)	116.2±45.1 (0.13)
	24	125.9±54.8 (<0.05)	161.9±72.4 (<0.05)	88.2±36.8 (0.26)	131.9±80.4 (0.04)
	36	161.9±66.3 (<0.05)	209.4±84.1 (<0.05)	127.7±38.9 (<0.05)	157.3±46.1 (<0.05)
	48	178.5±57.4 (<0.05)	218.8±74.6 (<0.05)	168.3±39.5 (<0.05)	216.1±50.5 (<0.05)

The roughness was analyzed in two randomly selected small areas of 6×6 µm^2^ and 3×3 µm^2^ to avoid any artifact caused by the cell structure. P value corresponds to the statistical results between the control (0 h) and the paclitaxel treated cells (6 h–48 h).

P-value is the statistical result between the nontreated cell and taxol treated cell.

**Figure 4:**
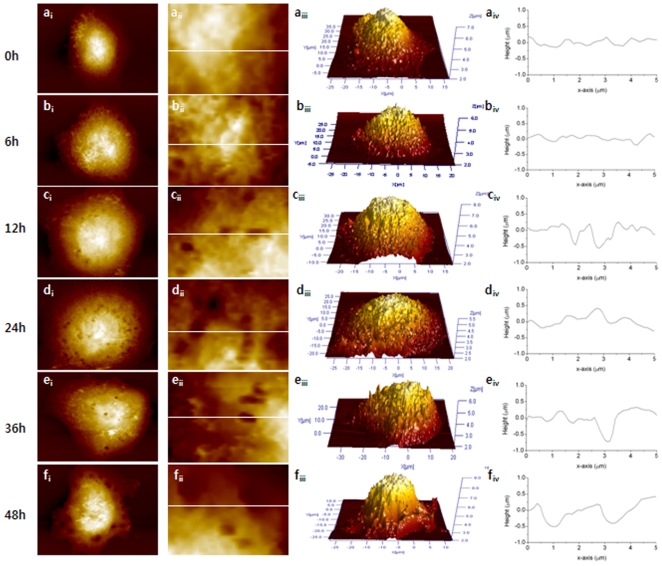
Representative AFM images from both untreated (0 h) and treated HeLa cells for 6, 12, 24, 36, and 48 h. The first and second columns show full and magnified images (3×5 µm^2^) of the cell. The third column shows a 3D image of the entire cell and the fourth column is the line profile measured in the magnified image shown in the second column. The white line shows the position of the line profile. The colors in the images indicate different heights with the light and dark colors corresponding to a higher and lower topography, respectively.

Figure 4 shows AFM images of HeLa cells; full (first column), magnified (second column), 3D images (third column) and line profiles (fourth column). All images were obtained under the same conditions as for the Ishikawa cells. The untreated HeLa cells were almost circular in shape with a smooth edge. The morphological changes induced by paclitaxel were similar to those observed in the Ishikawa cells. The cells that has been treated for 6 h showed no significant changes but cells treated for more than 12 h showed damage to their cell membranes. Compared to the Ishikawa cells, the membrane damage was less significant and the holes were smaller in number and size and its depth was shallower, as shown in Table 1. The mean height of the HeLa cells showed no dependence on the paclitaxel treatment. The surface roughness of HeLa cells was also increased by the paclitaxel treatment due to the damaged morphology, but it was a gradual change compared to the Ishikawa cells.

### 4. Effects of paclitaxel on cell membrane stiffness

**Figure 5:**
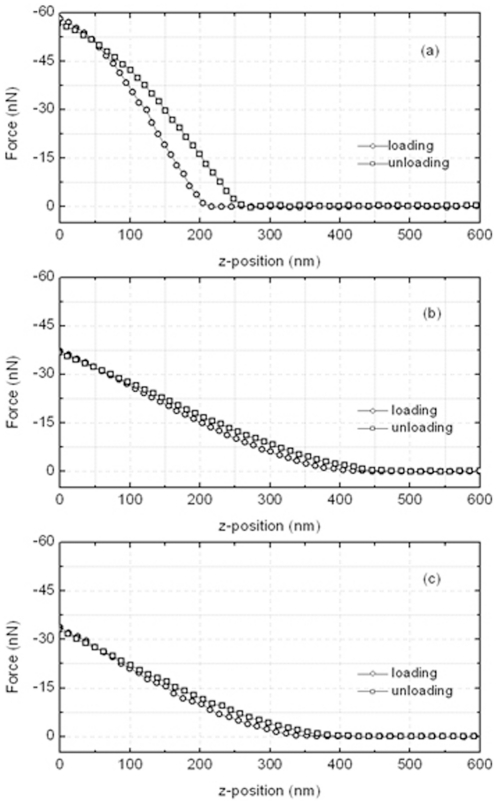
Force distance curve for control (a), treated Ishikawa (b) and HeLa (c) cells. The force of cantilever was plotted as a function of the probe position in the *z*-direction. The open circle and rectangle show the loading and unloading processes, respectively. The loading curve after the AFM tip approached closely or contacted the sample surface, which gives the information of the sample property.

The FD curve using AFM was obtained by measuring the amount of cantilever deflection as the probe approaches or retracts to the sample. Figure 5 shows the FD curves as a plot of the change in force, which is the multiply cantilever deflection by the spring constant of the cantilever, verses the probe position in the *z*-direction. Figs. 5(a), (b) and (c) are the FD curves of the control, treated Ishikawa (h = 6) and HeLa (h = 6) cells, respectively. The open circle and open rectangle represent the loading and unloading processes, respectively. The slop of the loading curve gives the information about the cell stiffness. The slop was largest in the control, and it decreased in both paclitaxel treated cells.

**Figure 6:**
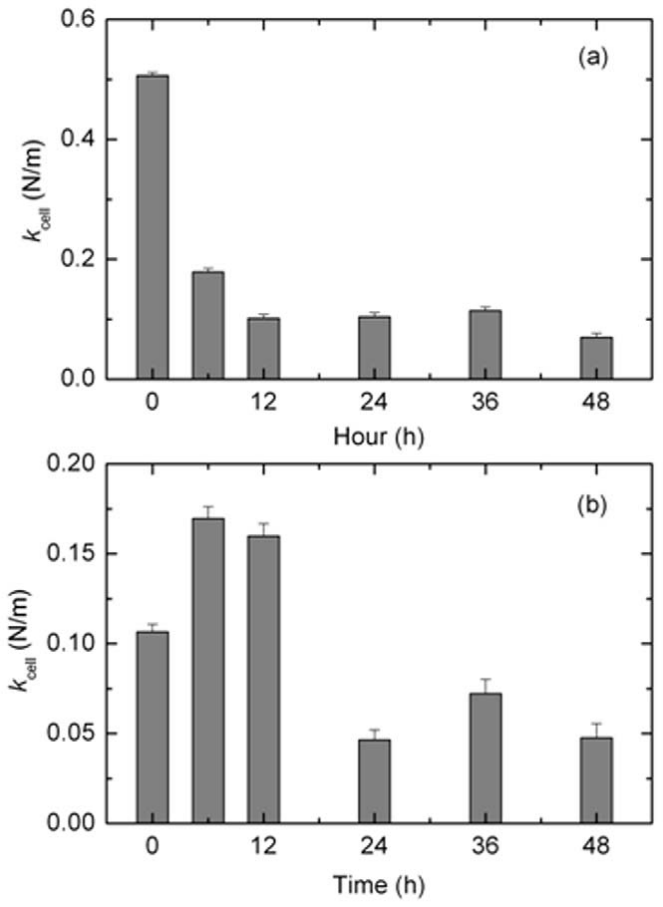
Cell membrane stiffness as a function of the paclitaxel treatment time in Ishikawa (a) and HeLa cells (b).

Figures 6(a) and (b) show the stiffness as a function of paclitaxel treatment time in Ishikawa and HeLa cells, respectively. The stiffness of the Ishikawa cells was significantly decreased by the paclitaxel treatment. The stiffness of the cells treated for 6 h was only 35% of that of the untreated cells. The stiffness decreased slightly at 12 h but there was no significant change after 12 h. In the HeLa cells, different changes in stiffness were observed. The stiffness of the treated cells for 6 and 12 h was larger than that of the untreated cell. In contrast, it decreased by 43∼67% for treatments longer than 24 h.

## Discussion

Although paclitaxel has demonstrated a wide spectrum of antineoplastic activity in a range of cancer cell types, the precise mechanism of apoptosis is unclear [1]–[6]. In the present study, the action of paclitaxel was examined by analyzing its effects on the morphology and microphysical properties of Ishikawa and HeLa cells. AFM showed that paclitaxel induced cell membrane damage in both cells. The membrane was damaged significantly after a 24 h paclitaxel treatment and the damage was more severe in Ishikawa cell than in HeLa cells. In both cells, the membrane was softer and rougher in the treated cells compared to those in the untreated cells. The MTT and TUNEL assays revealed the Ishikawa cells to be more sensitive to paclitaxel than HeLa cells, and the apoptosis was more apparent after 24 h treatment. These results are similar to the AFM results.

The MTT and TUNEL assay results showed the inhibitory effects of paclitaxel on cell proliferation according to the varying concentrations and treatment times. According to these results, although significant increases in TUNEL–positive cells appeared in both Ishikawa and HeLa cells after 24 h, the Ishikawa cells were more sensitive to paclitaxel than HeLa cells. This different response to paclitaxel between the two cell lines might be a result of the different organ originations and the extent of a reaction according to the tumor type. The antiproliferative activity of paclitaxel might be related to mitotic arrest or in part to either cytotoxic properties or the apoptotic process due to micromolar dosing [13]–[15]. The proliferation and apoptotic analyses suggest that a treatment with paclitaxel promotes apoptotic cell death and decreases in the proliferation of Ishikawa and HeLa cells.

Apoptosis is a physiological mode of cell death that removes cells at a given time or in response to a given stimulus in the absence of an inflammatory response. The process is defined by the unique morphological and biomechanical characteristics including cell shrinkage and nuclear condensation. Although there have been other characteristics attributed to the apoptotic process, the loss of volume or cell shrinkage is the single characteristic of programmed cell death observed in endometrial cancer cells [16]. Cisplatin, which is one of the primary drugs used in the treatment of cancers of the cervix, endometrium and ovaries, induces two different modes of cell death, necrosis and apoptosis [17]. Necrosis is characterized by cytosolic swelling and the early loss of plasma-membrane integrity, whereas apoptosis includes cell shrinkage and DNA fragmentation. Wang et al. reported that human lung adenocarcinoma cells swelled due to the paclitaxel treatment, resulting in increased cell height and decreased surface roughness [11]. On the other hand, in the paclitaxel-treated Ishikawa and HeLa cells, these characteristics of necrosis and apoptosis, including swelling or shrinkage, had not been observed previously [18]. In the present study, the typical apoptotic process was not observed in all paclitaxel-treated cells. Although the surface morphology of treated cells appear more convoluted than those of the untreated cells, the membrane surface still appears quite smooth and firm indicating no shrinkage. In both cells, the surface roughness increased with increasing treatment time but the cell heights did not showed any dependency on the paclitaxel treatment. The increased roughness can be understood by the damaged membrane not by cell shrinkage. As mentioned previously, the surface roughness is defined as the arithmetic mean of the deviations in height from the mean value [12]. Therefore, the holes in the membrane surface cause increased roughness, and the surface becomes rougher as the holes become deeper.

The holes on the membrane caused by paclitaxel are quite interesting but the mechanism is still unclear. The occurrence of macroscopic holes may be not the main path of apoptosis but could be the path of necrosis. Wang et. al. suggested that the holes observed in human lung adenocarcinoma cells may have resulted from paclitaxel-induced ER swelling, which was localized around the nucleus [11]. In the human lung adenocarcinoma cells, the holes were first observed after a 6 h paclitaxel treatment and the large holes appeared around the nucleus after 12 h treatment. Panayiotis et. al suggested that the holes appeared in the Ishikawa cell as a result of a deficit of the lamina [2]. The lamina gap was even observed in the cells in the process of exciting mitosis but never detected in the untreated cells. In addition, the lamina deficits were detected not only in cells treated with paclitaxel but also in the cells treated with toxotere and vinorelbine. Another way to produce holes on the membrane may be understood by the mechanism of action of paclitaxel in cancer cells. Paclitaxel causes damage to the mitotic spindle assembly, chromosome segregation and cell division [19]. Ovarian cancer cells were treated with paclitaxel resulted in the appearance of cytoplasmic vacuoles [20]. Paclitaxel can stabilize the microtubles, which results in an enhancement of microtubule polymerization, and can protect against microtubule disassembly and inhibit spindle formation in mitosis [21], [22]. The inhibited spindle functioning can suppress the microtubule dynamics at low concentrations (5–30 nM), and microtubule detachment from centrosomes can be suppressed at higher concentrations (<0.2 µM) [23], [24].

Recently, Sarah et al. performed an AFM study suggesting that cancer cells are more than 70% softer than normal cells. Therefore, the nanomechanical properties of a cell can be used as a cancer-detection method [25]. Depending on this report, it was assumed that the nanomechanical properties of the cancer cell may also be changed by antineoplastic drugs. If so, these properties will help in an evaluation of the anticancer activity of a drug. As expected, the Ishikawa and HeLa cells treated with paclitaxel were softer than the untreated cells, and the stiffness decreased with increasing treatment time. In both cell types, the paclitaxel treated cells were softer than the untreated cells except for HeLa cells treated for 6 and 12 h. The elasticity of a cell is related mainly to the intrinsic properties of the cell membrane and components of the cytoskeleton, such as microtubules, intermediate fibers and actin fibers. According to Antin et al., paclitaxel causes a reduction of actin filaments in myoblasts or excludes actin from interdigitation in microtubule-myosin arrays [26]. Owing to the paclitaxel treatment, the amounts of β-actin and laminin were reduced and the intensities of fibronectin and laminin in the cells was also reduced [27], [28]. Therefore, the changes in cell stiffness show good agreement with the paclitaxel-mediated activation of apoptosis in both cells.

In summary, this is the first report of an investigation of AFM-detected apoptotic morphological and biophysical changes in Ishikawa and HeLa cells after a paclitaxel treatment. Apoptotic cell death was induced by a paclitaxel treatment in both cell types. AFM was used to evaluate quantitatively the morphological changes in the cancer cells treated with paclitaxel. The changes in cell rigidity caused by paclitaxel were also revealed using FD curve measurements. The cell viability and apoptosis analyzed by a MTT and TUNEL assay showed good agreement with the AFM results. In conclusion, this study suggests that a study of the morphological and biophysical changes in cancer cells using AFM could be a potential method for evaluating the anticancer activity of a drug.

## Materials and Methods

### 1. Cells culture and treatments

Ishikawa cells was derived from well-differentiated human endometrial epithelial adenocarcinomas cell line(99040201; ECACC, UK) and grown in Minimum Essential Medium (Gibco, Auckland, New Zealand) containing 5% FBS (Sigma, USA), antibiotics (100 U/ml 122 of penicillin and 100 µg/ml of streptomycin) and 2 mM glutamate. HeLa cell was derived from human cervical cancer cell(KCLB-10002; KCLB, Korea), maintained in RPMI 1640 (WelGENE, Daegu, S. Korea) supplemented with 10% fetal bovine serum (Sigma, USA) and cultured on plastic substrata (SPL, USA) in a humidified 5% CO_2_ atmosphere at 37°C. The cells (2×10^5^) were seeded in dishes. Paclitaxel diluted in DMSO (dimethyl sulfoxide) was applied at 37°C for four different times. After the treatment, the cells were rinsed with serum-free medium, incubated for 2 h, and fixed.

### 2. Sample fixation

The cells were fixed by immersion in 2.5% gluteraldehyde for 1 hour for the AFM measurement or for 1 hour in methanol for the TUNEL assay. The cells were then rinsed three times with PBS and stored at room temperature in the dark.

### 3. MTT assay

The cell viability and proliferation were analyzed using a methylthiazol tetrazolium (MTT) assay. The colorimetric assay is based on the ability of live cells to reduce the yellow MTT reagent (Sigma, St Louis, MO, USA) to a purple formazan product. The cells were seeded in 12 wells (SPL, Tissue Culture Testplate) at various times (0, 6, 12, 24, and 48 h) and treated with paclitaxel (P4394, Sigma, USA) at various doses (10, 25, 50, and 75 µM). A total of 100 µl of a MTT solution was added to each well, and the cells were then incubated at 37°C and 5% CO_2_ for 1 h. After incubation, MTT was aspirated and 100 µl per well of DMSO was added to each well. Subsequently, the cell viability was assessed by measuring the absorbance at 540 nm.

### 4. TUNEL assay

For the detection of neuronal apoptosis, the terminal deoxynucleotidyl transferase (TdT)-mediated dUTP nick end-labeling (TUNEL) assay was performed in both Ishikawa and HeLa cells using a commercial kit (TACS® 2 TdT-Fluor *In Situ* Apoptosis Detection Kit) according to the manufacturer's instructions. Briefly, the cells were immersion hydrated, and the fixed and immobilized samples were rinsed with a PBS solution for 10 minutes. The samples were pre-incubated with a 2% proteinase K solution for 15 minutes at room temperature before being incubated in a TdT labeling buffer for 5 minutes and with a labeling reaction mix for 60 minutes in a humidity chamber. After the cells were washed with PBS (0.05%), they were incubated for 30 min in the dark with a Strep-Fluor solution. After an additional washing period, the tissues were covered with a DAPI mounting medium (Vector, #H-1200) and analyzed by fluorescence microscopy with a 495 nm filter.

### 5. Atomic force microscopy

The AFM system, NANOStation II (Surface Imaging Systems, Herzogenrath, Germany), consisted of an AFM scanner and Zeiss optical microscope (Epiplan 500×). The cancer cell images were measured with a reflex-coated gold cantilever in contact mode (Budget Sensor, Bulgaria). The material properties and dimensions of the probe used in contact mode were as follows: resonance frequency of 13 kHz (±4 kHz), force constant of 0.2 N/m (±0.14 N/m), cantilever length of 450 µm (±10 µm), cantilever width of 38 µm (±5 µm), cantilever thickness of 2 µm (±1 µm), tip radius of 5 nm (±1 nm), and tip height of 17 µm (±2 µm). All images were taken in a PBS buffer solution at a resolution of 256×256 pixels and a scan speed of 0.2 line/s. The scan area depended on the size of the cancer cell and ranged from 40×40∼60×60 µm^2^. To minimize the elastic effects in the high-resolution images, the samples were scanned at a low imaging force of <2 nN [29]. Image processing and data analysis were performed using SPIPTM software (Scanning Probe Image Processor version 4.1, Image Metrology, Denmark).

### 6. Surface roughness

The mean surface roughness (Ra) is defined as the arithmetic mean of the deviations in height from the line mean value, and Rq is the root mean square [12]. Since the roughness has a dependence on the sampling size, Ra and Rq were calculated for two different areas: 15 randomly selected 36 µm^2^ (6×6 µm^2^) and 20 randomly selected 9 µm^2^ (3×3 µm^2^) sections of the cell membrane. The holes were included in the roughness measurement. Statistical analysis using a two-tailed Student's t-test was applied to all pairs of samples (untreated-treated cells) to determine the significant differences in Ra and Rq. P-values<0.05 were considered significant.

### 7. Stiffness

The cell stiffness was examined using the force-distance curve (FD) obtained by measuring the amount of cantilever deflection as the probe was approached or retracted from the sample. The sample surface image was first scanned to determine the appropriate site for the FD curve without defects or impurities. The FD curves were measured in contact mode using the same cantilever as used for the image scan. To reduce the error originating from the cantilever and tip, all measurements were performed using the probes manufactured under the same conditions. The FD curve was measured more than 40 times per cell, more than 150 times for each treated groups. The loading rate of the probe was 1 µm/s. The slope of the FD curve after contact (approaching the curve) was used to determine the stiffness of the cell. To quantify the cell stiffness, the cellular spring constant of *k*_cell_ was calculated by modeling the cell-tip interaction as two springs [30] as follows:
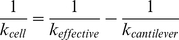
(1)where *k*_effective_ is the slope of the linear region of the FD curve for a cell, and *k*_cantilever_ is determined from each cantilever using a clean culture dish containing PBS.
